# In silico prediction of B cell epitopes of the extracellular domain of insulin-like growth factor-1 receptor

**Published:** 2016-12

**Authors:** Vahid Bayrami, Mehrnaz Keyhanfar, Hassan Mohabatkar, Manijeh Mahdavi, Violaine Moreau

**Affiliations:** 1 Department of Biotechnology, Faculty of Advanced Sciences and Technologies, University of Isfahan, Isfahan, Iran; 2 Department of Pharmaceutical Biotechnology, Faculty of Pharmacy and Pharmaceutical Sciences, Isfahan University of Medical Sciences, Isfahan, Iran; 3 Centre de Biochimie Structurale, CNRS UMR 5048, UM 1 & 2- INSERM U 1054, 29 rue de Navacelles, 34090, Montpellier, France

**Keywords:** IGF-1R, Cancer therapy, B cell epitope, Bioinformatics, Monoclonal antibody

## Abstract

The insulin-like growth factor-1 receptor (IGF-1R) is a transmembrane receptor with tyrosine kinase activity. The receptor plays a critical role in cancer. Using monoclonal antibodies (MAbs) against the IGF-1R, typically blocks ligand binding and enhances down-regulation of the cell-surface IGF-1R. Some MAbs such as cixutumumab are under clinical trial investigation. Targeting multiple distinct epitopes on IGF-1R, might be an effective strategy to inhibit IGF-1R pathway in cancer. In this study, new linear B cell epitopes for the extracellular domains of IGF-1R were predicted by in silico methods using a combination of linear B cell epitope prediction web servers such as ABCpred, Bepired, BCPREDs, Bcepred and Elliprro. Moreover, Discotope, B- pred and PEPOP web server tools were employed to predict new conformational B cell epitopes. In contrast to previously reported epitopes from extracellular region of the IGF-1R, we predicted new linear P8: (RQPQDGYLYRHNYCSK) and conformational Pc4: (HYYYAGVCVPACPPNTYRFE), Ppc6: (KMCPSTGKRENNESAPDNDT) and Ppc20: (ANILSAESSDSEFMQEPSGFI) epitopes. These epitopes are useful for further study as peptide antigens to actively immune host animals to develop new MAbs. Furthermore, the epitopes can be used in peptide-based cancer vaccines design.

## INTRODUCTION

Human Insulin-like growth factor 1 receptor (IGF-1R) is a tyrosine kinase receptor which mediates actions of insulin-like growth factor 1 (IGF-1) [1]. Research and clinical studies have indicated that IGF-1R and its ligands, insulin-like growth factors 1 and 2 (IGF-1 and IGF-2) and insulin have crucial role in the development, maintenance and progression of cancer [2].

Insulin receptor (IR) and IGF-1R share 70% sequence identity. Moreover, IGF-1, IGF-2 and insulin bind to the both receptors [3, 4]. The IGF-1R is a transmembrane and heterotetrameric protein consisting of two polypeptide chains; each chain has an extracellular, ligand-binding α-subunit and an intracellular β-subunit which exhibits tyrosine kinase activity [5].

The extracellular region can be sorted into 6 separate protein domains as follows: N-terminal receptor L domain (L1), cysteine-rich repeat domain (CRR), second receptor L domain (L2), and 3 fibronectin type III domains denoted as FnIII-1, FnIII-2, and FnIII-3 [3, 6, 7]. The IGF-1R over expression in the cancers often correlates with malignancy. This makes the receptor an attractive target for cancer immunotherapy [8].

One of the prevalent strategies to inhibit IGF-1R is the use of MAbs against the extracellular region of the receptor that hinders ligands binding and induces receptor internalization and degradation by endocytosis. However, due to the 70% identity between insulin receptor and the IGF-1R, the MAbs need to be specific inhibitors of the IGF-1R. To date, approximately 31 MAbs for the IGF-1R have been introduced and some of them are in different phases of clinical development [9, 10]. Among them, clinical antibody candidates, such as IMC-A12 (cixutumumab) and BIIB022, inhibited the IGF-1R signalling by blocking the IGF-1 and in some cases the IGF-2 binding and even causing IGF-1R down regulation [11-13]. Although most of these antibodies can inhibit tumor cell proliferation and growth, in vitro and in vivo, with differences in their mechanisms of action, some of them not only did not show any inhibiting effects but also increased ligand binding and stimulated tumor cell growth [10]. Furthermore, there are also some concerns that hyperglycemia can be a potential factor of increased patients’ morbidity. In phase I testing of cixutumumab, ganitumab and figitumumab on some cancer patients, these MAbs exhibited a toxicity profile with hyperglycemia as the most frequent adverse effect [14-16]. These results urged researchers to conduct more investigations and to develop novel humanized recombinant MAbs for the IGF-1R.

The IGF-1R is also considered as a target for vaccine development for primary prevention of murine model of breast cancer. Active immunotherapy with the peptide vaccines which are designed to be chimeric with multi-epitopes of B cells and T helper cells can induce generation of an adaptive immune response [17]. Several experimental techniques are currently available for selection of suitable B cell epitopes. The experimental approaches applied for detecting immunogenic regions are often laborious and resource-intensive. Computational techniques are fast, scalable, and cost-effective for B cell epitopes prediction, for focusing experimental investigations and for better understanding of antigen-antibody interactions [18-20]. Recent researches have shown there are limitations for the current epitope prediction methods. Hence, enhancing ther eliability of computational B cell epitope prediction methods remains a major challenge in computational vaccinology [21]. Nevertheless, prediction results produced by multiple computational tools could be used to gain a consensus result. Basically, the recognition of either small discrete T-cell epitopes or large conformational epitopes recognized by soluble antibodies and B cells, is the key molecular event for the immune response to pathogens [22]. B cell epitopes can be classified into two types: linear (continuous) and conformational (discontinuous). While linear epitopes comprise residues that are continuous in the sequence, conformational epitopes are composed of amino acids that are not neighboring in primary sequence and are brought into close proximity in the folded protein structure [23]. Localization of these epitopes is of clinical interest for the development of diagnostic tools, vaccines and cancer immunotherapies [24]. Many attempts have been made for predicting the antigenic sites from certain features of proteins primary structures. Different parameters such as static accessibility, hydrophilicity and mobility of the short segments in polypeptide chains have been associated with the position of continuous epitopes in proteins [25, 26]. The majority of MAbs against the IGF-1R bind to the overlapping surfaces on the cysteine-rich repeat (CRR) and the L1 and L2 domains [10]. Since the alanine scanning and biochemical studies have indicated that residues important for binding IGF-1 and IGF-2 to IGF-1R are located in the L1 and CRR domains [27, 28], most of the antibodies bind to this region with the competitive mechanism. There are accumulating evidences showing that targeting distinct multiple inhibitory epitopes on the IGF-1R and using a combination of competitive and allosteric antibodies may be more effective ways of affecting the IGF- 1R pathway in cancer [7, 29]. The IGF-1R specific IgG antibodies significantly rise in early-stage breast cancer patients at the time of diagnosis comparing to volunteer donors. Predicted T-helper epitopes, derived from the IGF-1R extracellular and transmembrane domains, induced a significantly greater incidence of Th2 immunity in breast cancer patients as compared to the controls. In addition, the magnitude of Th2 immunity was higher in breast cancer patients compared to volunteer donors. However, in contrast, both breast cancer patients and controls showed a similar incidence of Th1 immunity to IGF-1R domains with the predominant response directed against epitopes in the intracellular domain of the receptor [30].

The aim of present research is to predict new B cell epitopes for the IGF-1R extera- cellular region and particularly for the fibronectin domain. We predicted the 3D structure of fibronectin domain because there was no experimentally solved structure for the domains. Combination approaches were used by combining results from the sequence and the structure based methods and the solvent accessible surface area calculating tools. Furthermore, we used PEPOP application to predict new conformational epitopes from PDB structure of 3 N-terminal extracellular domains of the receptor (L1-CRR-L2).

## MATERIALS AND METHODS

**Linear B cell epitopes prediction for IGF-1R extracellular domains: **For prediction of linear epitopes, the sequence of extracellular region of IGF-1R was submitted to ABCpred, BCPREDS, Bcepred, Bepipred and Ellipro servers. The hidden Markov model, Thornton's method, Support Vector Machine classifiers, Recurrent Neural Network and physico-chemical properties of amino acids were applied to predict linear B cell epitopes [31-35]. Only, the linear peptides which were predicted frequently by 3 or more servers were selected.

**Homology modeling of type III fibronectin domains of IGF-1R: **Crystallographic structure of 3 N-terminal extracellular domains of the IGF-1R (L1-CRR-L2) is determined by x-ray crystallography, however since to date there is no experimentally solved structure for the fibronectin domains. Hence, in this study for prediction of conformational epitopes for type III fibronectin domains, a PDB structure of the domains was predicted by homology modeling. The FASTA format of amino acid sequence related to IGF-1R (accession number: P08069) was obtained from UniProt database (http://www.uniprot.org/). The sequence was submitted to I-TASSER server [36] to find the appropriate template with sufficient query sequence coverage and the sequence identity. Crystallographic structure of the template for homology modeling (PDB ID: 2DTG chain E) was obtained from PDB database (http://www.pdb.org). The final complete model was generated using Modeller v9.11 [37]. The energy level of the final model was minimized using 3Drefine server [38]. The predicted model was evaluated using Q-mean [39] and ProSA servers [40] by Qmean and z scores respectively.

**Conformational B cell epitope prediction for IGF-1R: **DiscoTope 2.0 and B-pred servers were used for prediction of conformational epitopes from the entire PDB structure of receptor that obtained from the homology modeling method. The DiscoTope method incorporates a new spatial neighborhood description and a half- sphere exposure as a surface measure based on the protein structure and epitope propensity scoress and predicts residues that can be involved in B-cell epitopes [41]. B- pred is a web-based platform for scoring and predicting B-cell epitopes based on the structures of the potential immunological proteins. The method scores the peptides set of a protein based on the average solvent exposure, by a filter on filtering the average local model quality for each peptide [42]. Solvent accessible areas on the PDB structures are calculated using Naccess program V2.1.1 [43]. Relative solvent accessibility of predicted peptides were calculated using NetSurfP ver. 1.1 sever [44]. Default settings were applied to all the tools used. The conformational peptides that were predicted using both Discotope and B-pred servers with high Relative Solvent Accessibility (RSA) score were used for further studies.

PEPOP, a server web based application [45], was also used for prediction of potential conformational epitopes from PDB structure of 3 N-terminal extracellular domains of receptor (L1-CRR-L2) with PDB ID 1IGR. PEPOP uses the 3D structure of a protein to predict clusters of the solvent exposed segments according to their spatial proximity. The segments are then combined to yield peptides that should correspond to the discontinuous epitopes. PEPOP methods define a set of segments which is combined (the path between the segments) starting from one segment (the reference segment). A set of segments is close segments either included in a cluster or in a 10Å-radius patch on the surface of the protein. In the current study, five PEPOP methods were used: the optimized nearest neighbor (ONN), the optimized flanking nearest neighbor (OFN), the optimized patched segments path (OPP), the SHortest Path based (SHPnat) and the Traveling Salesman Problem based (TSPnat) methods [46]. ONN, OFN and OPP find the optimized path corresponding to the arrangement of the segments having the shortest total distance. SHP and TSP are graph-based methods which model a protein with nodes representing the segments which can only be added in their natural sense and edges being weighted by Euclidian distances. These methods respectively use SHP and TSP algorithms to find the optimal path between the segments. Relative solvent accessibility was also calculated using NetSurfP ver. 1.1 sever for peptides, predicted by PEPOP.

PEPOP systematically was run on the 3 N-terminal extracellular domains. Peptides with less than 14 amino acids and more than 22 amino acids were removed for reducing the large number of produced peptides. The peptides having several long segments (> 3 amino acids) predicted using several methods were kept. The relative solvent accessibility was also calculated using NetSurfP ver. 1.1 sever for the peptides, predicted by PEPOP.

**Sequence Alignment of predicted peptides with insulin receptor: **The development of MAbs against the IGF-1R is a complex process due to the homology between the extracellular domain of the IGF-1R and the IR. Due to the identity between the insulin receptor and the IGF-1R, the MAbs need to bind specifically only to the IGF-1R. Extracellular domains of the IGF-1R are only 53% identical to the IR extracellular domains, which have enabled development of anti-IGF-1R antibody inhibitors which are known noncross-reactive. Hence, in this study the amino acid sequence of the predicted peptides was aligned against human insulin receptor using ClustalW2 Multiple Sequence Alignment tool (http://www.ebi.ac.uk/Tools/msa/clustalw2/). The results were manually checked to obtain the best peptides with less identity to the IR.

**Determining the sequences with glycosylation site: **Amino acids in glycosylated regions may be shielded from presentation to an antibody by masking carbohydrates [47]. Therefore, among the predicted epitopes, any sequence that contains glycosylation site must be discarded. The glycosylation sites on the extracellular region of the receptor were retrieved from UniProt database (http://www.uniprot.org/) and the predicted peptides containing glycosylated residues were removed from the results and were not used for further study.

## RESULTS

**Figure1:**
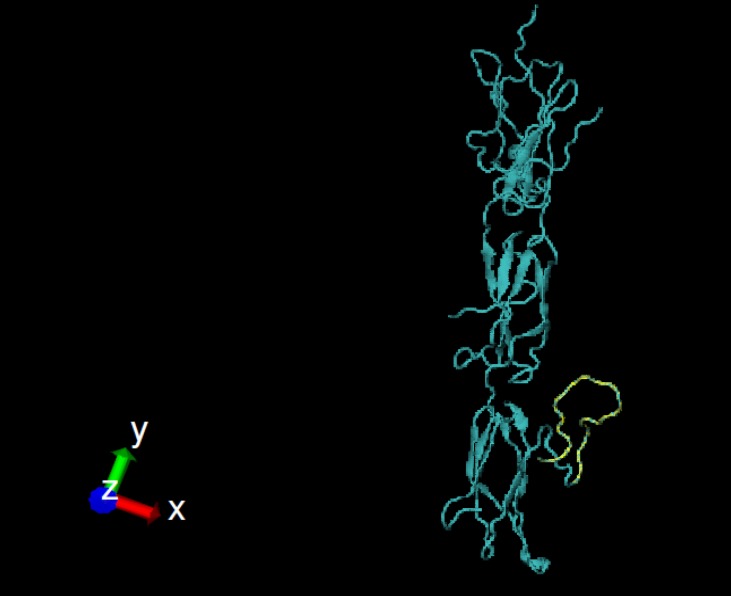
Homology model for the type III fibronectin domains of the IGF-1R. P8 linear peptide is shown in yellow. The picture was generated using V.M.D 1.9.1

The predicted linear B cell epitopes of the IGF-1R are shown in Table 1. As listed in the table, peptides P4 and P8 are predicted by 5 servers used in the current study. P4 has low (33%) and P8 has no identity with the IR respectively. These peptides reside in 251- 266 and 650-665 regions of the receptor shown in Fig 1.

**Table 1:** The predicted 16mer linear B-cell epitopes

Peptide	Sequence	Position	Server^a^	Identity^b^
**P1 **	LCPGTMEEKPMCEKTT	181-196	1,2,4,5	NO
**P2 **	RCQKMCPSTCGKRACT	210-225	1,2,4	53%
**P3 **	STCGKRACTENNECCH	217-232	1,2,4	40%
**P4 **	CRHYYYAGVCVPACPP	251-266	1,2,3,4,5	33%
**P5 **	KGDINTRNNGERASCE	474-489	1,2,5	50%
**P6 **	HFTSTTTSKNRIIITW	494-509	1,3,4,5	20%
**P7 **	RGAKSEILYIRTNASV	595-610	1,4,5	57%
**P8 **	RQPQDGYLYRHNYCSK	650-665	1,2,3,4,5	NO
**P9 **	DGTIDIEEVTENPKTE	675-690	1,4,5	NO
**P10 **	CGGEKGPCCACPKTEA	692-707	1,3,4,5	36%
**P11 **	FLHNSIFVPRPERKRR	725-740	1,3,4,5	43%

ABCpred(1), Ellipro(2), Bcepred(3), BepiPred(4), BCPred(5)-

(a) Servers that predicted the corresponding B-cell epitopes or part of the epitope are numbered as:

(b) Identity with human insulin receptor

Using automated mode of I-TASSER, a primary model was predicted for the IGF- 1R based on chain E of the insulin receptor structure (2DTG) as template with 85% query coverage and 55% identity. As a result, 94% of the query was modeled using this server and the 6% misaligned regions were completed using loop-modeling performed by Modeller v9.11. The 3Drefine server was used for structure minimization of the output model. In addition, Qmean server was used for model quality assessment. In the assessment, the best crystallographic structures needed to obtain a Qmean score close to 1 and the score of the final model was 0.73. The output z-score for the final model, calculated by ProSA server, was -8.26. This score indicated that the predicted model had the X-ray quality. The homology model for the type III fibronectin domains of the IGF-1R is shown in Figure 1.

Discotope and B-pred predicted 8 peptides corresponding to conformational epitopes or part of four epitopes, approximately located in the region of linear B cell epitopes (Pc1, Pc2, Pc4, and Pc6 shown in Table 2).

**Table 2:** Predicted conformational B Cell epitopes by Discotope and B-pred

**Peptide** a	**Sequence**	**Position**	**RSA** b	**Identity** c
**Pc1**	PKECGDLCPGTMEEKPMCEK	175-194	0.467	57%
**Pc2**	CGDLCPGTMEEKPMCEKTTI	178-197	0.436	55%
**Pc3**	GTMEEKPMCEKTTINNEYNY	184-203	0.465	45%
**Pc4**	HYYYAGVCVPACPPNTYRFE	253-272	0.42	NO
**Pc5**	DSEGFVIHDGECMQECPSGF	292-311	0.419	56%
**Pc6**	QRQPQDGYLYRHNYCSKDKI	649-668	0.444	33%
**Pc7**	KQAEKEEAEYRKVFENFLHN	709-728	0.456	59%
**Pc8**	SRNTTAADTYNITDPEELET	754-773	0.416	32%

(a) Conformational Peptides are showed by (c) i. e Pc1-

(b) Relative Solvent Accessibility-

(c) Identity with human insulin receptor

**Figure 2:**
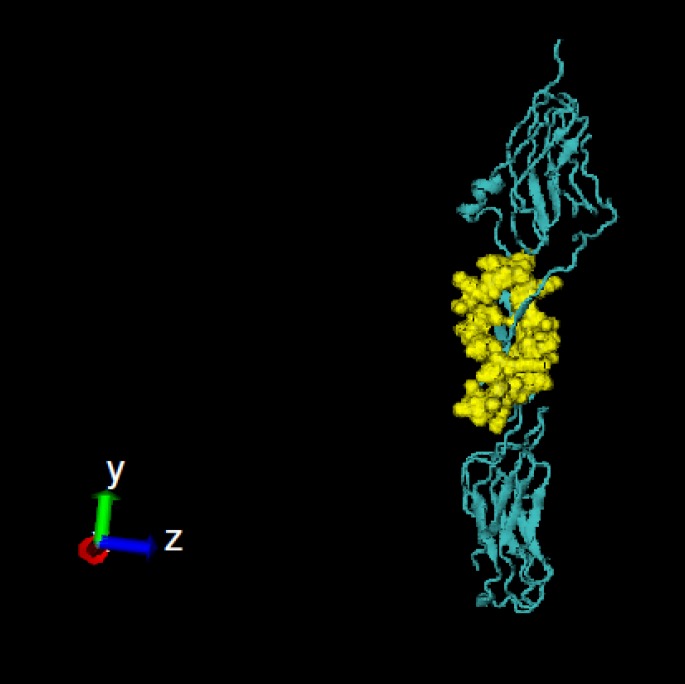
Pc4 conformational B cell epitope on fibronectin III domains of the IGF-1R is shown as yellow spheres. The picture was generated using V.M.D 1.9.1

Further analysis for solvent accessible areas and relative solvent accessibility of all the residues on the PDB structures using Naccess program and NetSurfP server defined that predicted conformational B cell epitopes had higher solvent accessible and their residues were exposed on the surface (Table 2). These are important factors for immunogenicity of an antigen or synthetic peptide. Among these conformational epitopes, only Pc4 had no identity with human insulin receptor (Table 2 and Fig. 2).

**Figure 3:**
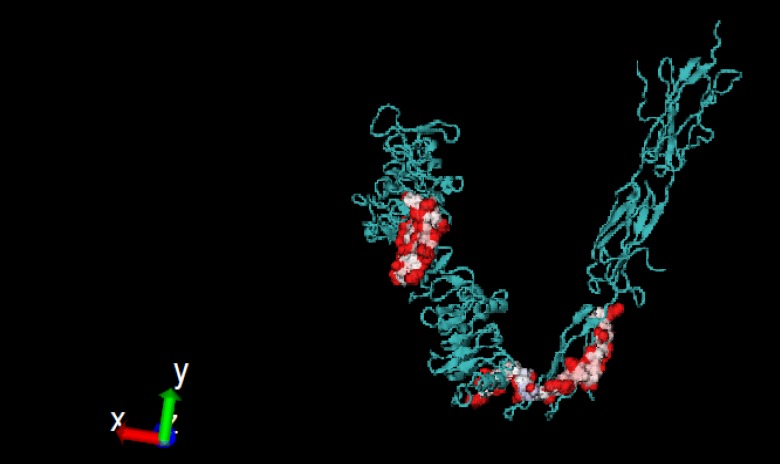
Conformational Ppc6 and Ppc20 peptides, on the IGF-1R extracellular domains, are shown as spheres. The picture was generated using V.M.D 1.9.1

In the current study, the 5 most appropriate optimized methods among the 34 methods predicting discontinuous peptides in PEPOP were used [46]. PEPOP was systematically run on the 3 N-terminal extracellular domains: each segment has been used at a turn as the reference segment to predict a peptide. Thus, all the possible peptides were predicted. Among the 386 predicted peptides, the peptides of less than 14 amino acids and more than 22 amino acids were removed and 104 peptides remained. Among the 104 peptides, the peptides predicted using more than one method were kept and for the rest (predicted using only one method), peptides including 3 segments of more than 3 amino acids were also kept (9 peptides). As a result, 20 peptides were automatically designed (Table 3). Conformational peptides (Ppc20 and Ppc6), predicted with high Relative Solvent Accessibility (RSA) score and no identity with human IR are shown in Figure 3.

**Table 3:** PEPOP-predicted conformational epitopes

**Peptide** a	**Sequence**	**Length**	**RSA** b	**Identity** c
Ppc1	TINNEYNYTNRCKMCPST	18	0.42	NO
Ppc2	RHYYYAGVPACPPNDRDF	18	0.42	33%
Ppc3	PSGFINGSQSMYIPEGPCPKV	21	0.41	52%
Ppc4	PACPPNDRDFANILSAESSDSE	22	0.4	NO
Ppc5	KMCPSTENNESAPDNDT	17	0.45	NO
Ppc6	KMCPSTGKRENNESAPDNDT	20	0.46	NO
Ppc7	TNRCENNESAPDNDTCVT	18	0.4	50%
Ppc8	PACPPNDRDFFMQEPSGFI	19	0.41	NO
Ppc9	TNRCENNESAPDNDTCVTNPK	21	0.46	38%
Ppc10	TMEEKPMEKTINNEYNYTNRC	21	0.45	NO
Ppc11	TNRCTMEEKPMEKTINNEYNY	21	0.4	50%
Ppc12	PSGFIIPEGPCPKVNGSQSMY	21	0.43	57%
Ppc13	KMCPSTGKRHPENNESAPDNDT	22	0.42	50%
Ppc14	TNRCKETNSKAEDYRSYR	18	0.45	NO
Ppc15	GDLTNRCKMCPSTGKRHP	18	0.4	33%
Ppc16	GDLTNRCTMEEKPMEK	16	0.41	NO
Ppc17	TMEEKPMEKTINNEYNYS	18	0.41	50%
Ppc18	KGDLTMEEKPMEKSTNRC	18	0.42	35%
Ppc19	KMCPSTGKRHPRHYYYAGV	19	0.42	50%
Ppc20	ANILSAESSDSEFMQEPSGFI	21	0.46	NO

a) ( PEPOP predicted Conformational epitopes are showed by (pc) i.e. Ppc1-

(b ) Relative Solvent Accessibility-

(c) Identity with human insulin receptor

## DISCUSSION

Although the majority of B cell epitopes appear to be conformational, most of the computational methods focused on the prediction of sequential epitopes [20]. Linear epitope prediction approaches can be classified as propensity scale methods, improved propensity scale methods and machine learning methods [48]. If the tertiary structure of an antigen is known, there are improved methods for identifying conformational B cell epitopes. Examples are Discotope web server and PEPOP. These are based on features like amino acid propensity scales and solvent accessibility [41, 45].

In this study, linear and conformational B cell epitopes of the IGF-1R were predicted using both primary sequence and tertiary structure. Based on combination approaches and considering lowest identity with the IR and frequently prediction using several tools, the best peptides were the linear B cell epitope P8 (Table 1 and Fig. 1) and conformational B cell epitopes Pc4 (Table 2 and Fig. 2). Experimental epitope mapping of some other MAbs against the IGF-1R such as 9E11 (241-266), 7C2 (241-266), αIR-3 (223-274) and 24-60 (184-283) showed overlapping epitopes with P4 (251-266) [28, 49]. Linear epitope P8 (650-665) and conformational epitope Pc6 (649-668) were approximately in the identical region of fibronectin domain. In addition, PEPOP also predicted 20 new conformational peptides (Table 3). The predicted epitopes by PEPOP are widely distributed within the CRR domain and often partly overlapped, consistent with the view that PEPOP predicted segmented epitopes and the CRR domain displayed a mosaic of overlapping epitopes. Considering less identity with the IR and high RSA score (Table 3) conformational epitopes, Ppc6 and Ppc20 can be suitable for further experimental tests (Fig. 3).

Previous biochemical research projects have shown that both inhibitory and agonistic epitopes exist within the first 4 extracellular domains of the IGF-1R [7]. The first and most well characterized antibody to the IGF-1R, αIR-3, was developed in mice. This is an inhibitory antibody and binds to the cysteine-rich repeat domain, residues between 223 and 274, and specifically blocks the IGF-1 binding to the IGF-1R, while having only a weak effect on the binding of the IGF-2 [50]. Many of the IGF-1R residues which are involved in the IGF-1 and the IGF-2 binding have been characterized using the alanine-scanning mutagenesis. These studies have indicated residues 240-241- 242-251 and 266 in the CRR domain which are important for the IGF-1 binding but not for the IGF-2 binding to the IGF-1R [27, 28]. Based on the epitope mapping studies, binding of the IGF-1 and the IGF-2 to the IGF-1R can be decreased by blocking residues across the region of the IGF-1R covering both CRR and L2 domains. This region seems much larger than a single antibody epitope [51]. In the present work, a new conformational epitope Pc4 (HYYYAGVCVPACPPNTYRFE/253-272) and a linear B cell epitope P4 (CRHYYYAGVCVPACPP/251-266) and the covering part of the CRR domain were predicted using various bioinformatics analyses. Based on the previous studies [7], antibody binding to these epitopes may lead to the receptor down regulation and the inactive form of the IGF-1R.

In a study, using chimeric IGF-1R/IR constructs, it was shown that an inhibitory epitope existed in the FnIII-1 domain [52]. In addition, another study identified that one class of allosteric IGF-1 and IGF-2 blockers bound to a separate epitope on the outer surface of the IGF-1R FnIII-1 domain [7]. Based on the distance of the epitope from the ligand binding pocket of the receptor, this region is obviously an allosteric surface. Binding of an antibody to FnIII-1 could lead to conformational changes in the IGF-1R and may damage the ligand binding. In the present work, new conformational B cell epitopes Pc6, Pc7 and Pc8 (Table 2) in FnIII-1 domain were predicted (using Discotope and B-pred servers). Due to the lowest identity with the IR and the high relative solvent accessibility, conformational peptides Pc6 and Pc8 are appropriate candidates for production of MAbs and targeting the IGF-1R allosteric domain.

Dong et al. [29] showed that combining two inhibitory IGF-1R antibodies with distinct epitopes and ligand-blocking mechanisms could direct to a greater inhibition of the receptor signaling. In addition, the tumor growth can be inhibited through enhanced ligand blockade and receptor down regulation. Using a combination of allosteric and competitive inhibitors for more efficient ligand blockade, such as MAbs against the epitopes reported in this paper, could be a method to overcome any potential limitations with single IGF-1R antibody and possibly to provide improved clinical efficacy. With these new epitopes in hand, further studies are needed to correlate these fine epitopes specificity evolution of both allosteric and competitive inhibitor MAbs against the IGF- 1R. Many theoretical and experimental efforts have been conducted to understand the relation between the construction of a protein and its immunogenic properties. For instance, several potential antigenic linear epitopes were identified in a B subtype strain of envelope glycoprotein of HIV-1 (IIIB) using Preditop computer program [18]. Epitopes and structural properties of Iranian HPV-16 E6 were also predicted using bioinformatic methods [53]. In another study, linear and conformational B cell epitopes of the HER 2 ECD-Subdomain III were predicted using in silico methods [54].

In conclusion, findings of the present work, using the bioinformatics analyses could be used in MAb production, cancer therapy, vaccine design and the diagnostic tools. In addition, the current in silico approaches, are reducing time and minimizing the total number of necessary tests to find possible and proper epitopes. In the next step, synthesis of determined peptides, in vitro and in vivo experimental studies are essential for assurance of the predicted epitopes.

In these experiments, identification of epitopes on the IGF-1R, that are either stimulatory or inhibitory, is very important for development of strategies to better manipulation of the IGF-1R responses for therapeutic benefits. Overall, selection of suitable epitopes of the IGF-1R as antigens, and utilizing them for raising MAbs against the IGF-1R, with ability of cancer inhibition would be beneficial in cancer treatment. To the best of our knowledge, for the first time, in this study the linear and conformational B cell epitopes of the IGF-1R extracellular domains were predicted, screened and assessed using the well-known bioinformatics comprehensive analyses.
